# Antiviral drug valacyclovir treatment combined with a clean feeding system enhances the suppression of salivary gland hypertrophy in laboratory colonies of *Glossina pallidipes*

**DOI:** 10.1186/1756-3305-7-214

**Published:** 2014-05-08

**Authors:** Adly MM Abd-Alla, Carmen Marin, Andrew G Parker, Marc JB Vreysen

**Affiliations:** 1 Insect Pest Control Laboratory, Joint FAO/IAEA Division of Nuclear Techniques in Food and Agriculture, International Atomic Energy Agency, Wagramer Straße 5, A-1400 Vienna, Austria

**Keywords:** Diptera, Glossinidae, Salivary gland hypertrophy virus, *Hytrosaviridae*

## Abstract

**Background:**

*Hytrosaviridae* cause salivary gland hypertrophy (SGH) syndrome in some infected tsetse flies (Diptera: Glossinidae). Infected male and female *G. pallidipes* with SGH have a reduced fecundity and fertility. Due to the deleterious impact of the virus on *G. pallidipes* colonies, adding the antiviral drug valacyclovir to the blood diet and changing the feeding regime to a clean feeding system (each fly receives for each feeding a fresh clean blood meal) have been investigated to develop virus management strategies. Although both approaches used alone successfully reduced the virus load and the SGH prevalence in small experimental groups, considerable time was needed to obtain the desired SGH reduction and both systems were only demonstrated with colonies that had a low initial virus prevalence (SGH ≤ 10%). As problems with SGH are often only recognized once the incidence is already high, it was necessary to demonstrate that this combination would also work for high prevalence colonies.

**Findings:**

Combining both methods at colony level successfully suppressed the SGH in *G. pallidipes* colonies that had a high initial virus prevalence (average SGH of 24%). Six months after starting the combined treatment SGH symptoms were eliminated from the treated colony, in contrast to 28 months required to obtain the same results using clean feeding alone and 21 months using antiviral drug alone.

**Conclusions:**

Combining valacyclovir treatment with the clean feeding system provides faster control of SGH in tsetse than either method alone and is effective even when the initial SGH prevalence is high.

## Findings

Tsetse flies (*Glossina* spp.) are the vectors of sleeping sickness in humans (human African trypanosomosis), and the cattle disease nagana [1,2]. Nagana is considered among the greatest constraints to livestock production in sub-Saharan Africa. The prophylactic and curative treatment of livestock with trypanocidal drugs is problematic due to the development of resistance of the parasites to the available trypanocidal drugs. Therefore, removal of the vector, the tsetse fly, is considered the most efficient way of managing nagana [3,4].

There are a number of efficient tsetse control tactics available that can be combined and applied in the frame of the area-wide integrated pest management approach. One of these tactics is the sterile insect technique (SIT), which is very efficient for controlling and eliminating low-density populations previously reduced with other control methods [5]. Using this approach, a successful sustainable eradication of *Glossina austeni* from Unguja Island (Zanzibar), United Republic of Tanzania, was achieved [6]. Consequently, other programmes were developed to apply this approach on the African mainland and, in 1996, the Government of Ethiopia embarked on such a programme to create a zone free of *Glossina pallidipes* in the Southern Rift Valley of Ethiopia [7,8]. In this programme the establishment and expansion of *G. pallidipes* colonies for mass-production of sterile male flies proved to be difficult and several colony collapses were experienced. The cause of these failures was identified as the salivary gland hypertrophy virus (GpSGHV), which was often present at high prevalence and affects colony productivity and stability [9].

The virus transmission mode in wild tsetse populations is most probably mother to offspring transmission, either trans-ovum or through infected milk glands. In laboratory-maintained flies where blood feeding using an *in vitro* membrane feeding system is used, horizontal transmission was found to be the most important route of virus transmission. In order to develop GpSGHV management strategies several studies were conducted on various aspects of the biology of the virus [10-13]. Following these basic studies, several virus management approaches were suggested [14] and the effect of two of these strategies on the prevalence of SGH in colonies of *G. pallidipes* was recently published i.e. adding the antiviral drug valacyclovir to the blood diet of the flies [15,16] and the application of a clean feeding strategy (each fly received a clean fresh blood meal each time) [17,18]. Briefly, the clean feeding system can be initiated in a large-scale colony by selecting as many cages of teneral flies from the main colony as can be fed at one time on the available clean membranes and thereafter these flies and their progeny should always be fed first on the fresh, clean blood (that has not been supplied to other flies). These flies will, over time develop into a clean feeding colony, and when the virus reaches a very low level (no more hypertrophy) so that contamination of the blood is minimal, a second set of cages of teneral flies is started in the same manner, fed second on the membranes now containing essentially no virus. This process is continued group by group until all the flies are kept in these separate groups. Both clean feeding and the addition of valacyclovir successfully reduced the virus load and the prevalence of SGH symptoms in the treated *G. pallidipes* groups when implemented separately. More rapid results were obtained when these approaches were combined with selection of low virus prevalence at a small scale experimental level [18]. Despite the successful reduction of the SGH prevalence in these colonies, both approaches had some obvious limitations i.e. (i) the long time needed after treatment initiation to achieve significant reduction in virus load (21 months with valacyclovir and 28 months with clean feeding system) and (ii) both approaches were only demonstrated for groups with a low initial virus load and low SGH prevalence (≤10%). In operational large scale production facilities, the detection of virus problems usually surfaces when a lower productivity of the colony becomes apparent, usually when the prevalence of the virus is already high. In such cases, the main objective is to restore the productivity of the colony (i.e. reduce the virus load) in the shortest possible time to prevent any interruption of the operational program. We therefore tested the impact of the combined approaches in large scale colony level on a high virus prevalence colony.

All experiments were carried out with the colony described previously by Abd-Alla *et al.*[19], kept in the same manner. Previous analysis revealed that ca 4–10% of individuals in this colony showed SGH [18]. A high SGH prevalence colony (SGH of 24%) was developed by exposing flies to the virus by feeding blood contaminated with the virus. Flies from this colony were used to start two new experimental colonies: the first to be maintained under a clean feeding system combined with valacyclovir treatment (the flies were fed on fresh blood supplemented with valacyclovir (300 μg/ml)) [18], the flies of the second colony were fed on contaminated blood. The blood for this colony was first used for two feeding rounds of flies of the main colony, then the blood was collected from the feeding trays and valacyclovir was added to the required concentration (300 μg/ml) and used for feeding the experimental flies. The prevalence of SGH was determined by dissection of flies (n = 360) from different ages.

The graphics were created using the ggplot2 package [20] in R [21]. Dissection results were analysed by analysis of variance (ANOVA/ANCOVA) in R [20] after arcsine square root transformation.

**Figure 1:**
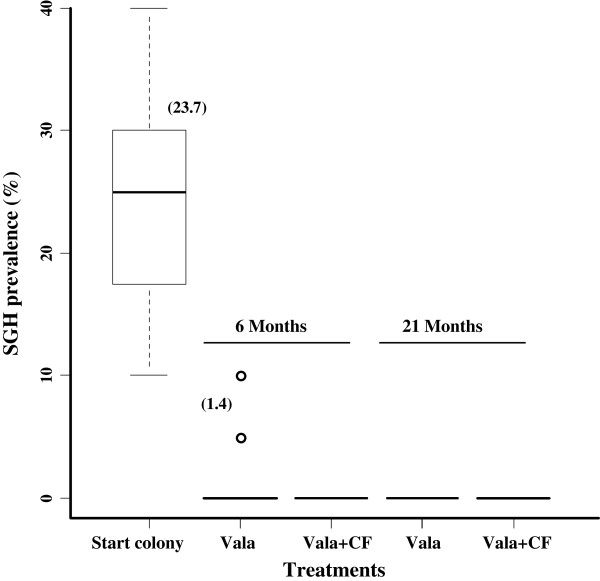
**Effect of the combination of valacyclovir and clean feeding system on SGH prevalence in the Seibersdorf *G. pallidipes *Tororo colonies by fly dissection.** Flies (n = 360) of different ages were randomly selected from the different colonies at two time points after implementation of the clean feeding system and dissected to determine the status of the salivary glands. Numbers between brackets are the mean percentage SGH prevalence. Vala: valacyclovir, CF: clean feeding. There is no significant difference between any of the treatments except the initial prevalence.

The dissection results presented in Figure 1 show that the prevalence of the SGH syndrome in the clean feeding system with valacyclovir treatment was significantly reduced (F_2,61_ = 93.989, *P* < < 0.001) after six months i.e. from 24% (the average prevalence in the colony before treatment [21]) to 0%, irrespective of the age of the flies (F_1,64_ = 0.914, *P* = 0.343). In the valacyclovir treatment with contaminated feeding, the prevalence of SGH (1.4%,) was significantly reduced after six months (F_1,24_ = 73.304, *P* < < 0.001) but the SGH was only eliminated from the treated flies after 21 months. Although the difference in the prevalence of SGH between the valacyclovir treatment implemented alone or combined with clean feeding at 6 or 21 months was not significant (F_1,52_ = 2.634, *P* = 0.111), the antiviral drug without the clean feeding system required 21 months post treatment to achieve complete elimination of SGH syndrome from the treated colony.

Taken together, the data presented in this paper demonstrate that the clean feeding strategy to reduce the virus load and to remove the SGH syndrome in colonies of *G. pallidipes* can be improved by combining this system with a valacyclovir treatment. The time needed to eliminate the SGH syndrome from the colony was reduced from 28 months [19] to 6 months, leading to sustainable maintenance and expansion of the colony. The data together with Abd-Alla *et al.*[19] also confirm that the implementation of the feeding regime in combination with valacyclovir treatment is readily applicable in tsetse mass rearing facilities and only requires changes to the colony handling and recording system, which only needs minimal additional training of the staff at no significant additional cost to the SIT program compared to the cost of other methods to manage the virus. Also, once the prevalence of SGH has been reduced to zero, antiviral drug treatment may be withdrawn to reduce the cost so long as the clean feeding system is strictly observed. The data presented in this article strongly supports the implementation of a clean feeding strategy in combination with valacyclovir treatment in large-scale *G. pallidipes* production facilities in order to achieve sustainable GpSGHV control.

## Abbreviations

GpSGHV: Glossina pallidipes salivary gland hypertrophy virus; SGH: Salivary gland hypertrophy; SIT: Sterile insect technique.

## Competing interests

The authors declare that they have no competing interests.

## Authors’ contributions

Conceived and designed the experiments: AMMA, AGP and MJBV. Performed the experiments: CM. Drafted the manuscript: AMMA, AGP and MJBV. All authors read and approved the final manuscript.

## References

[B1] WHO Control and surveillance of human African trypanosomiasis: report of a WHO expert committee (WHO technical report series; no. 984) 2013 Geneva

[B2] Steelman CD Effects of external and internal arthropod parasites on domestic livestock production Annu Rev Entomol 1976 21 155 178 10.1146/annurev.en.21.010176.001103 2093

[B3] Leak SGA Tsetse biology and ecology: their role in the epidemiology and control of trypanosomosis 1998 Wallingford: CABI Publishing

[B4] Jordan AM Trypanosomiasis control and African rural development 1986 London: Longman

[B5] Vreysen MJB Saleh KM Lancelot R Bouyer J Factory tsetse flies must behave like wild flies: a prerequisite for the sterile insect technique PLoS Negl Trop Dis 2011 5 1 4 10.1371/journal.pntd.0000907 PMC3042992 21364965

[B6] Hendrichs J Kenmore P Robinson AS Vreysen MJB Vreysen MJB, Robinson AS, Hendrichs J Area-Wide Integrated Pest Management (AW - IPM): Principles, Practice and Prospects Area-wide control of insect pests. From research to field implementation 2007 Dordrecht, The Netherlands: Springer 3 33

[B7] Feldmann U Dyck VA Mattioli RC Jannin J Dyck VA, Hendrichs J, Robinson AS Potential impact of tsetse fly control involving the sterile insect technique Sterile Insect Technique. Principles and Practice in Area-Wide Integrated Pest Management 2005 Dordrecht, The Netherlands: Springer 701 723

[B8] Alemu T Kapitano B Mekonnen S Aboset G Kiflom M Bancha B Woldeyes G Bekele K Feldmann U Vreysen MJB, Robinson AS, Hendrichs J Area-wide control of tsetse and trypanosomosis: Ethiopian experience in the Southern Rift Valley Area-Wide Control of Insect Pests: From Research to Field Implementation 2007 Dordrecht, The Netherlands: Springer 325 335

[B9] Abd-Alla A Bossin H Cousserans F Parker A Bergoin M Robinson A Development of a non-destructive PCR method for detection of the salivary gland hypertrophy virus (SGHV) in tsetse flies J Virol Methods 2007 139 143 149 10.1016/j.jviromet.2006.09.018 17070938

[B10] Sang RC Jura WGZO Otieno LH Mwangi RW The effects of a DNA virus infection on the reproductive potential of female tsetse flies, *Glossina morsitans centralis* and *Glossina morsitans morsitans* (Diptera: Glossinidae) Mem Inst Oswaldo Cruz 1998 93 861 864 10.1590/S0074-02761998000600030 9921317

[B11] Jura WGZO Otieno LH Chimtawi MMB Ultrastructural evidence for trans-ovum transmission of the DNA virus of tsetse, *Glossina pallidipes* (Diptera: Glossinidae) Curr Microbiol 1989 18 1 4 10.1007/BF01568821

[B12] Sang RC Jura WGZO Otieno LH Ogaja P Ultrastructural changes in the milk gland of tsetse *Glossina morsitans centralis* (Diptera; Glissinidae) female infected by a DNA virus J Invertebr Pathol 1996 68 253 259 10.1006/jipa.1996.0093 8954813

[B13] Feldmann U Ochieng’-Odero JPR Guidelines for the rearing of tsetse flies using the membrane feeding technique Techniques of insect rearing for the development of integrated pest and vector management strategies. Volume 1 1994 Nairobi, Kenya: ICIPE Science Press 449 471

[B14] Mutika GN Marin C Parker GA Vreysen MJB Boucias GD Abd-Alla AMM Impact of salivary gland hypertrophy virus infection on the mating success of male *Glossina pallidipes*: consequences for the sterile insect technique PLoS ONE 2012 7 e42188 10.1371/journal.pone.0042188 22912687 PMC3418267

[B15] Kariithi HM Ince AI Boeren S Vervoort J Bergoin M van Oers MM Abd-Alla A Vlak JM Proteomic analysis of *Glossina pallidipes* salivary gland hypertrophy virus virions for immune intervention in tsetse fly colonies J Gen Virol 2010 91 3065 3074 10.1099/vir.0.023671-0 20719992

[B16] Kariithi HM van Lent JW Boeren S Abd-Alla AM Ince IA van Oers MM Vlak JM Correlation between structure, protein composition, morphogenesis and cytopathology of *Glossina pallidipes* salivary gland hypertrophy virus J Gen Virol 2013 94 193 208 10.1099/vir.0.047423-0 23052395

[B17] Abd-Alla AMM Parker AG Vreysen MJB Bergoin M Tsetse salivary gland hypertrophy virus: hope or hindrance for tsetse control? PLoS Negl Trop Dis 2011 5 e1220 10.1371/journal.pntd.0001220 21912708 PMC3166039

[B18] Abd-Alla AMM Adun H Parker AG Vreysen MJB Bergoin M The antiviral drug valacyclovir successfully suppresses salivary gland hypertrophy virus (SGHV) in laboratory colonies of *Glossina pallidipes* PLoS ONE 2012 7 e38417 10.1371/journal.pone.0038417 22679503 PMC3367962

[B19] Abd-Alla AMM Karim EIA Mohamed A Lapiz E Parker AG Vreysen MJB Managing hytrosavirus infections in *Glossina pallidipes* colonies: feeding regime affects the prevalence of salivary gland hypertrophy syndrome PLoS ONE 2013 8 e61875 10.1371/journal.pone.0061875 23667448 PMC3646844

[B20] Wickham Hggplot2: elegant graphics for data analysis 2009 New York: Springer

[B21] R: A language and environment for statistical computing http://www.R-project.org

